# Three-Dimensional BiOI/BiOX (X = Cl or Br) Nanohybrids for Enhanced Visible-Light Photocatalytic Activity

**DOI:** 10.3390/nano7030064

**Published:** 2017-03-14

**Authors:** Yazi Liu, Jian Xu, Liqiong Wang, Huayang Zhang, Ping Xu, Xiaoguang Duan, Hongqi Sun, Shaobin Wang

**Affiliations:** 1 Department of Chemical Engineering, Curtin University, GPO Box U1987, Perth, WA 6845, Australia; yazi.liu@postgrad.curtin.edu.au (Y.L.); huayang.zhang@postgrad.curtin.edu.au (H.Z.); 2 School of Chemistry and Life Science, Nanjing University Jinling College, Nanjing 210089, China; xj_tank@163.com (J.X.); xupzerone@gmail.com (P.X.); 3 State Key Laboratory of Pollution Control and Resource Reuse, School of the Environment, Nanjing University, Nanjing 210046, China; wangliqiong168@163.com; 4 School of Engineering, Edith Cowan University, 270 Joondalup Drive, Joondalup, WA 6027, Australia; h.sun@ecu.edu.au

**Keywords:** BiOI/BiOCl, visible light, photocatalysis, heterojunction, degradation, water oxidation

## Abstract

Three-dimensional flower-like BiOI/BiOX (X = Br or Cl) hybrids were synthesized via a facile one-pot solvothermal approach. With systematic characterizations by X-ray diffraction (XRD), scanning electron microscopy (SEM), Transmission electron microscopy (TEM), the Brunauer-Emmett-Teller (BET)specific surface area, X-ray photoelectron spectroscopy (XPS), and the UV-Vis diffuse reflectance spectra (DRS), the BiOI/BiOCl composites showed a fluffy and porous 3-D architecture with a large specific surface area (SSA) and high capability for light absorption. Among all the BiOX (X = Cl, Br, I) and BiOI/BiOX (X = Cl or Br) composites, BiOI/BiOCl stands out as the most efficient photocatalyst under both visible and UV light irradiations for methyl orange (MO) oxidation. The reaction rate of MO degradation on BiOI/BiOCl was 2.1 times higher than that on pure BiOI under visible light. Moreover, BiOI/BiOCl exhibited enhanced water oxidation efficiency for O_2_ evolution which was 1.5 times higher than BiOI. The enhancement of photocatalytic activity could be attributed to the formation of a heterojunction between BiOI and BiOCl, with a nanoporous structure, a larger SSA, and a stronger light absorbance capacity especially in the visible-light region. The in situ electron paramagnetic resonance (EPR) revealed that BiOI/BiOCl composites could effectively evolve superoxide radicals and hydroxyl radicals for photodegradation, and the superoxide radicals are the dominant reactive species. The superb photocatalytic activity of BiOI/BiOCl could be utilized for the degradation of various industrial dyes under natural sunlight irradiation which is of high significance for the remediation of industrial wastewater in the future.

## 1. Introduction

In the past few decades, intensive research has been focused on the efficient utilization of solar energy as a promising and sustainable strategy to address the energy crisis and environmental contamination. Solar energy is a natural resource which is inexhaustible as well as environmentally-friendly [1,2,3]. Among the solar energy conversion and applications, such as photocatalytic decomposition of organic pollutants [4,5,6], solar cells [7], water splitting [8,9,10], as well as catalytic CO_2_ reduction [11,12,13], the rational development of efficient semiconductors and construction of optimal heterojunction nanocomposites to enhance the utilization of solar energy have turned out to be the two most effective techniques. Among the popular photocatalysts, TiO_2_ has been widely investigated due to its superb photocatalytic activity, good chemical stability, relatively low toxicity, and cost [14]. However, TiO_2_ can only absorb and respond to ultraviolet light due to its wide band gap (3.2 eV), which severely limits its practical applications in solar energy conversion. Recently, photocatalysts containing bismuth with high visible-light-induced activity have attracted considerable attention in designing novel photocatalysts, such as BiFeO_3_ [15], BiVO_4_ [16], Bi_2_MoO_6_ [17], Bi_2_WO_6_ [18], etc. Bismuth oxyhalides, BiOX (X = Cl, Br, I), have shown remarkable photocatalytic activity due to their unique structure and physiochemical properties [19,20,21,22]. The bismuth oxyhalides were comprised of a layered structure of [Bi_2_O_2_] slabs interleaved by double slabs of halogen atoms with an internal static electric field, which could facilitate the rapid separation of photo-generated electrons and holes. Among these new family members, BiOI shows the highest absorption capacity for visible light irradiation due to its relatively narrow band gap. However, the photocatalytic degradation of organics on pure BiOI is still unsatisfactory due to the high recombination rate of the photo-generated carriers, which constrains the photocatalytic activity for solar energy utilization.

Heterogeneous coupling has been adopted as a fantastic strategy to improve photocatalytic activity by minimizing the recombination rate of photogenerated carriers. Meanwhile, a nanoporous structure with a large surface area is especially attractive for heterogeneous photocatalysis due to the multiple scattering effects [23]. Three-dimensional microstructure hybrids fabricated from nanoscaled building blocks may further contribute to the enhancement of catalytic performance by providing abundant transport paths for the reactants to arrive at the active sites. For the hybrid catalysts constructed with a heterojunction, both of the semiconductors would adjust their bandgaps to obtain the composite valence band (VB) and conduction band (CB), which could be tuned to be more suitable for visible light excitation. However, the intrinsic mechanism of the photocatalytic process over BiOI/BiOX (X = Cl or Br) composites is still uncertain.

In this study, a simple one-pot solvothermal process was adopted to synthesize 3D BiOI/BiOX (X = Cl or Br) flower-like microspheres with a high specific surface area and superior visible light photo-absorption ability. The performances of the BiOI/BiOX (X = Cl or Br) composites and pure BiOX (X = Cl, Br, I) were evaluated by photo-oxidation of methyl orange (MO) under both UV and visible light irradiation. The BiOI/BiOCl composites also demonstrated an excellent activity for water oxidation under simulated solar light irradiation. In addition, the information about the composite band structure and the reactive oxygen species in the photocatalytic process were unveiled for an in-depth mechanistic study. The composite was finally assessed for practical remediation of versatile industrial dyes under natural sunlight irradiation.

## 2. Experimental

### 2.1. Materials

Bismuth (III) nitrate pentahydrate (Bi(NO_3_)_3_^.^5H_2_O) was purchased from Sinopharm Chemical Reagent Co. Ltd., Shanghai, China. Ethylene glycol (HOCH_2_CH_2_OH), potassium iodide (KI), sodium bromide (NaBr), potassium chloride (KCl), and ethanol (CH_3_CH_2_OH, absolute) were obtained from Nanjing Chemical Reagent Co. Ltd., Nanjing, China. Methyl orange and methyl violet were purchased from Shanghai Sansi Co. Ltd., Shanghai, China. Direct black 38 was purchased from Tokyo Chemical Industry Co. Ltd., Tokyo, Japan. 5,5-dimethyl-1-pyrroline N-oxide (DMPO, ≥98.0%) was purchased from Sapphire Bioscience Pty. Ltd., (Redfern, New South Wales, Australia). Silver nitrate (≥99.0%) and lanthanum (III) oxide (≥99.9%) was purchased from Sigma-Aldrich (Castle Hill, New South Wales, Australia). All the chemicals are analytical reagents and were received without further treatment.

### 2.2. Synthesis of BiOX (X = Cl, Br, I) 

The BiOX was synthesized via a simple solvothermal method. More specifically, 3.88 g Bi(NO_3_)_3_·5H_2_O and 1.33 g KI were each dissolved in a 40 mL ethylene glycol solution, separately. Then the Bi(NO_3_)_3_ solution was dropped into the KI solution gradually with vigorous stirring for 5 min using a magnetic stirrer on a hotplate to form a homogeneous mixture. Then the solution was transferred to a 120 mL Teflon-lined autoclave, sealed, and treated in an oven at 160 °C for 12 h and cooled down naturally. The precipitate was filtered and washed with deionized water and ethanol three times each, and finally dried in the oven at 60 °C for 24 h. BiOCl and BiOBr were prepared following the same protocol based on a mole ratio of Bi(NO_3_)_3_·5H_2_O:NaBr(KCl) = 1:1.

### 2.3. Synthesis of BiOI/BiOX (X = Cl, Br)

The BiOI/BiOCl and BiOI/BiOBr composites were prepared via a similar procedure according to a previous report with minor modifications [24]. In a typical procedure, 1.96 g Bi(NO_3_)_3_^.^5H_2_O was dissolved in 80 mL ethylene glycol with vigorous stirring for 30 min to form a transparent solution. Then, 0.6 g KI and 0.03 g KCl were added into the above solution and continued to stir for another 2 h. Afterward, the mixed solution was transferred to a 120 mL Teflon-lined autoclave, sealed, and treated in an oven at 160 °C for 12 h and cooled down naturally. The precipitate was filtered and washed with deionized water and ethanol three times each, and finally dried in the oven at 60 °C for 24 h. Thus, BiOI/BiOCl was obtained. The synthesis of BiOI/BiOBr followed the same procedure based on a molar ratio of KI:NaBr = 9:1.

### 2.4. Characterization Techniques

The crystal structures of the as-synthesized catalysts were characterized via X-ray diffraction analysis (XRD) with Cu Kα radiation in a X′TRA diffractometer (ARL Company, Swiss, Basel, Switzerland). Scanning electron microscopy (SEM, X650, Hitachi Company, Tokyo, Japan) and transmission electron microscopy (TEM, 200CX, JEOL Company, Tokyo, Japan) were employed to study the surface morphology and structure of the catalysts. The specific surface areas were measured using N_2_ adsorption/desorption isotherms on a Micromeritics ASAP 2020, and the pore size distribution was calculated from the desorption isotherm. The UV-visible diffuse reflectance spectra (DRS) were performed on a Shimadzu UV-2401 UV-Vis spectrophotometer equipped with an integrated sphere attachment. X-ray photoelectron spectroscopy with Al Kα X-ray radiation (PHI 5000 VersaProbe, ULVAC-PHI, Kanagawa, Japan) was adopted to investigate the surface elemental composition. Electron paramagnetic resonance (EPR) was performed on a Bruker EMX plus spectrometer (Bruker Company, Rheinstetten, Germany) under the conditions of modulation amplitude (8 G), modulation frequency (100 kHz), microwave frequency (9.48 GHz), and non-saturating microwave power (1.02 mW). DMPO was utilized as a chemical probe to capture the produced radicals from photocatalysis. 0.2 g/L BiOI/BiOCl was first mixed with a 40 μL DMPO solution. Then the mixed solution was extracted via the capillary and tested under both dark and simulated solar light irradiation after 5 min. The superoxide radicals (·O_2_^−^) were captured by changing the solution to methanol to quench the hydroxyl radicals.

### 2.5. Evaluation of the Photocatalysts

The photocatalytic activities were evaluated by the degradation of organic dyes in aqueous solution under UV light (CEL-LAX Xe lamp 300 W; UV cut-off filter <400 nm) with a light intensity of 268 mW/cm^2^ and visible light (CEL-LAX Xe lamp, 300 W; visible cut-off filter 350–680 nm) with a light intensity of 405 mW/cm^2^. Specifically, 0.05 g of photocatalyst was suspended into 250 mL MO solution (20 mg/L). Prior to irradiation, the suspensions were magnetically stirred in the dark for 1 h to ensure the establishment of an adsorption/desorption equilibrium. During the photocatalytic process, 3 mL of the reaction solution was extracted every 30 min and centrifuged at 13,000 rpm for 15 min to remove the particles. Then the concentration of the MO solution was measured by a UV-Vis spectrometer (Perkin-Elmer Lambda 900UV/Vis/NIR, Waltham, MA, USA) at the maximum absorbance wavelength of 465 nm.

The catalysts were also evaluated in a water oxidation process for oxygen evolution. The reaction was processed in a black jacket reactor with a 300 W Xenon lamp as the simulated solar light source. Silver nitrate was selected as the electron scavenger. In a typical procedure, 0.1 g of catalyst was added to 200 mL of solution including AgNO_3_ (0.03 M) and La_2_O_3_ (0.2 g). The suspensions were mixed under vigorous stirring for 30 min in the dark and degassed to remove the air prior to irradiation. The produced O_2_ was in situ analyzed by gas chromatography (Agilent 490 Micro GC, New South Wales, Australia) equipped with a thermal conductive (TCD) detector (Agilent 490 Micro GC, New South Wales, Australia).

## 3. Results and Discussion

### 3.1. Morphology and Structure

**Figure 1:**
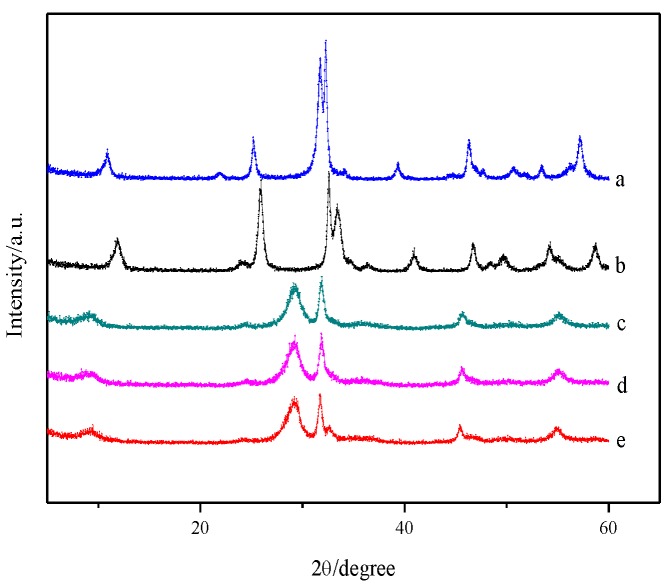
X-ray diffraction (XRD) patterns of (**a**) BiOBr, (**b**) BiOCl, (**c**) BiOI/BiOBr, (**d**) BiOCI/BiOl, and (**e**) BiOI.

Figure 1 shows the phase structures of the as-synthesized samples of pure BiOX (X = Cl, Br and I) as well as BiOI/BiOX (X = Cl and Br) composites. The diffraction peaks of a, b, and e in Figure 1 can be fully indexed to the tetragonal BiOBr phase (JCPDS card No. 78-0348), tetragonal BiOCl phase (JCPDS card No. 73-2060), and tetragonal BiOI phase (JCPDS card No. 73-2062) with no impurities. The BiOI/BiOCl and BiOI/BiOBr composites exhibit the characteristic peaks of pure BiOI whereas no obvious peaks for BiOCl and BiOBr were discovered, possibly due to their low loading amount. Comparing the profiles of d with e, it can be seen that all diffraction peaks slightly shift to the higher angles, corresponding to a smaller spacing distance between the different planes. The same phenomenon could be observed for BiOI/BiOBr. Moreover, the diffraction peaks of the composites are broader than the corresponding peaks of pure BiOI, indicating that the crystallite sizes of BiOI/BiOCl and BiOI/BiOBr become smaller during heterogeneous growth, which is in good accordance with the literature [25,26,27].

**Figure 2:**
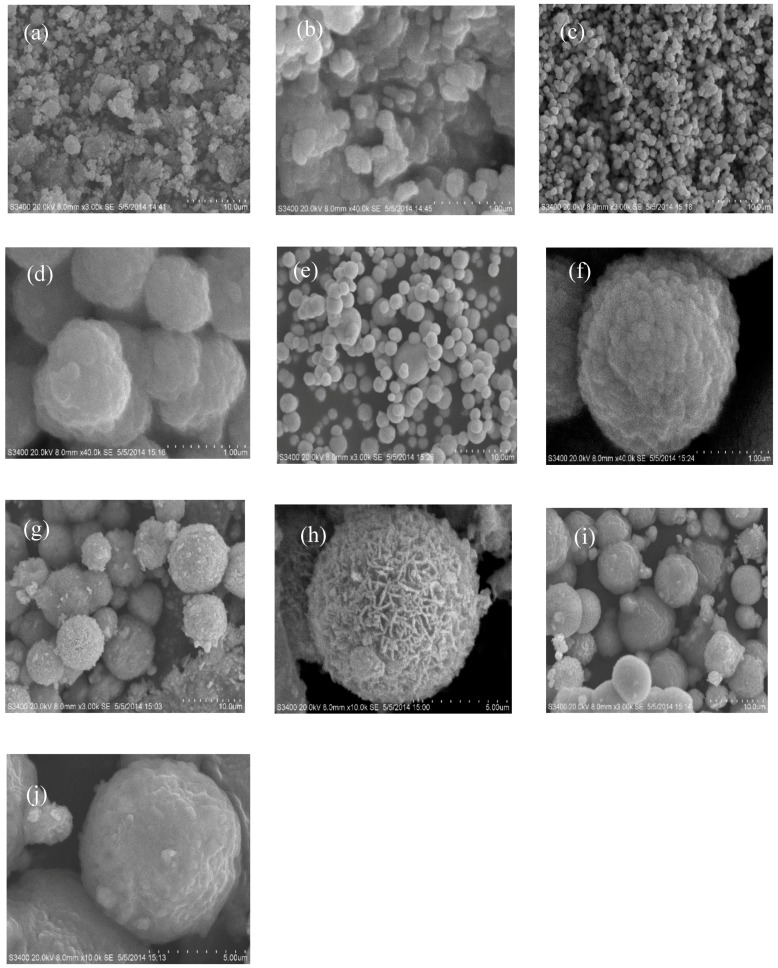
Scanning electron microscopy (SEM) images of (**a**,**b**) BiOI; (**c**,**d**) BiOCl; (**e**,**f**) BiOBr; (**g**,**h**) BiOI/BiOCl and (**i**,**j**) BiOI/BiOBr with different magnification levels.

The SEM images of the photocatalysts are presented in Figure 2. All the BiOX samples present as microspheres with different diameters, morphologies, and microstructures. Among them, BiOBr shows the largest particle size with a diameter between 1.5 and 2.1 µm, which is larger than that of BiOCl (0.4–1 µm) and BiOI (0.1–0.5 µm). High-magnification images in Figure 2b,d,f indicate that all the microspheres are composed of nanoplates with a thickness of 25 nm, which were aggregated at the core compactly. Moreover, the nanoplates in BiOI in Figure 2b were closely packed together to form irregular microspheres with no obvious gaps. For BiOCl, the microsphere particles are presented with regular shapes with multilayers in Figure 2d. The microspheres of BiOBr in Figure 2f are constructed of diverse diameters with regular shapes and large gaps. As seen from Figure 2g–j, the nanocomposites of BiOI/BiOX (X = Br, I) with diameters around 2–8 µm exhibit three-dimensional micro/nano-architectures aggregated by monolithic or monomeric particles, which are larger than the pure BiOX (X = Cl, Br, I). More interestingly, compared to the compact BiOI/BiOBr clusters, BiOI/BiOCl composites appear as more loose and fluffy agglomerates. The hierarchical 3D micro-hybrid with a porous structure may lead to a high specific surface area and surface-to-volume ratio, as well as abundant transport paths for charge-carrier separation, which could be favorable for a photocatalytic process [28].

**Figure 3:**
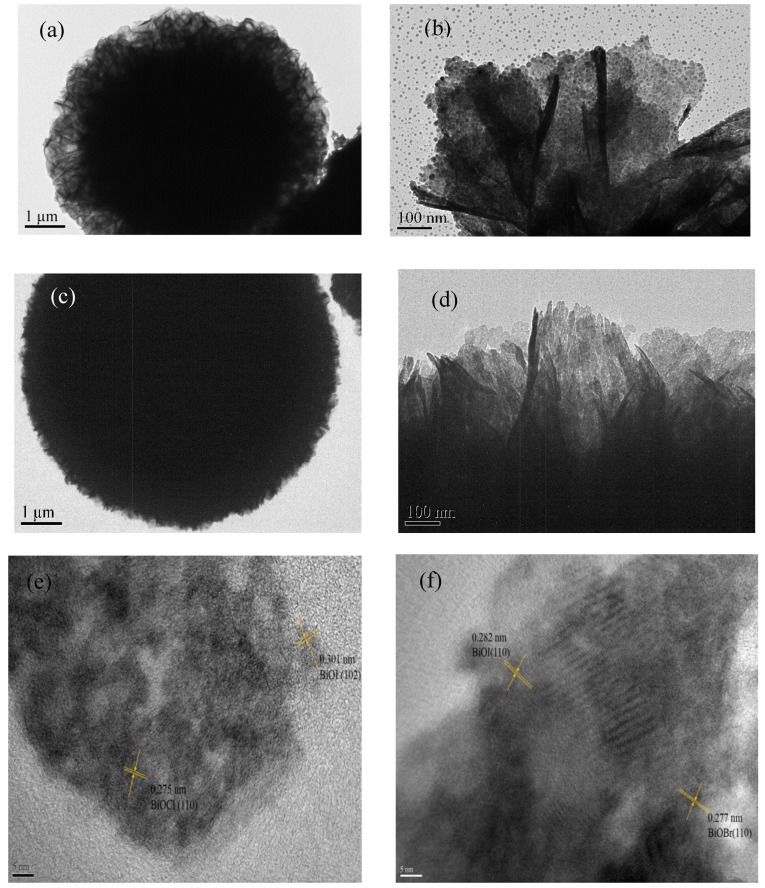
Transmission electron microscopy (TEM) images of (**a**,**b**) BiOI/ BiOCl; (**c**,**d**) BiOI/ BiOBr samples and high resolution transmission electron microscopy (HRTEM) images of (**e**) BiOI/BiOCl; (**f**) BiOI/BiOBr.

The TEM images further unveiled the morphological structure of the BiOI/BiOCl and BiOI/BiOBr composites. Figure 3a,c exhibits flower-like 3D architectures and BiOI/BiOBr show a more compact microstructure. Both of the nanoplates in Figure 3b,d are very thin with a thickness of around 20 nm. The TEM image in Figure 3b (BiOI/BiOCl) shows a more regular morphology with smaller particle size than that of BiOI/BiOBr in Figure 3d. In addition, two different lattices of BiOCl can be observed from the high-resolution transmission electron microscopy (HRTEM) image in Figure 3e with the d-spaces of 0.301 nm for the (102) plane of BiOI and 0.275 nm for the (110) plane. Figure 3f also exhibits two different crystalline structures with d-spaces of 0.282 nm and 0.277 nm, which can be assigned to the (110) plane of BiOI and the (110) plane of BiOBr, respectively. The structures of the composites revealed in the TEM images are in agreement with the SEM images.

### 3.2. Optical Properties

**Figure 4:**
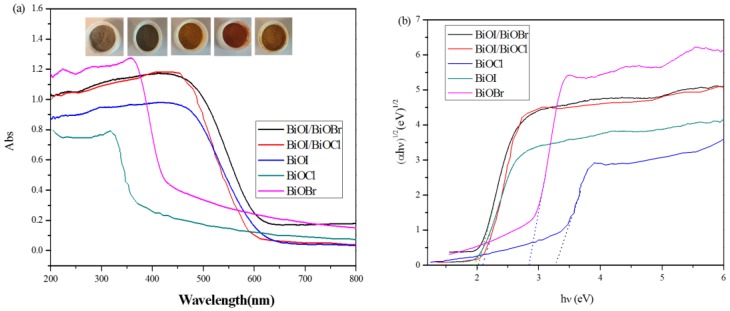
Ultraviolet-visible diffuse reflectance spectra (UV-Vis DRS) of (**a**) as-prepared samples and (**b**) the plotting of (α*h*γ)^1/2^ vs. photon energy.

The band gap structure and electronic states of a semiconductor photocatalyst are of crucial importance to determine the photoabsorption capacity and catalytic performance. Figure 4a displays the UV-Vis diffuse reflectance spectra of the BiOX (X = Cl, Br, I) as well as BiOI/BiOX (X = Br, Cl) composites. BiOCl is only responsive to UV light with an absorption edge at approximately 375 nm, while BiOI exhibits typical optical absorbance in the visible light region with the absorption edge at about 630 nm. BiOBr shows great absorption capacity in both UV and visible light regions with the absorption edge at 450 nm. Compared to BiOX, BiOI/BiOX (X = Br, Cl) composites exhibit enhanced photoabsorption capacity with a slight shift to the lower wavelength. The optical band gap energy can be evaluated based on the plots of (α*h*γ)^1/2^ vs. photon energy [24,26] shown in Figure 4b. By extrapolating the linear portion of the plots to zero, the band gap energy *E_g_* of BiOCl, BiOBr, BiOI, BiOI/BiOCl, and BiOI/BiOBr were estimated to be 3.25, 2.85, 1.93, 2.09, and 2.01 eV, respectively, with the color transition from off-white to brick red with the red shift of the bandgap (Figure 4a, insert). The results are consistent with previously reported values [25,29,30]. Meanwhile, the band gap of BiOI/BiOCl is located between BiOI and BiOCl due to the formation of a solid solution, which is also observed for BiOI/BiOBr.

### 3.3. XPS Analysis

**Figure 5:**
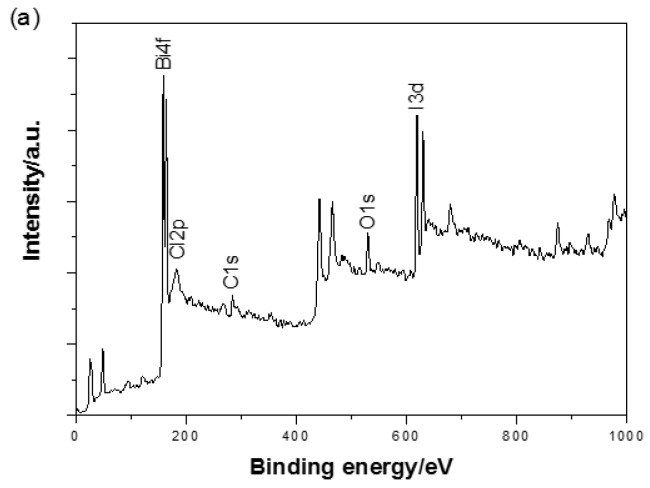

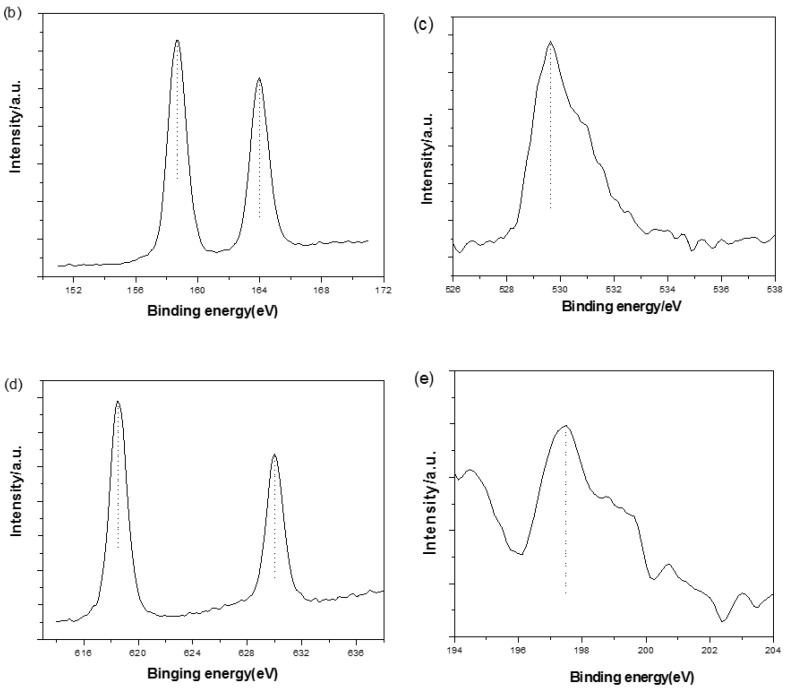
(**a**) X-ray photoelectron spectroscopy (XPS) spectra survey and high-resolution scan of (**b**) Bi 4f; (**c**) O 1s; (**d**) I 3d and (**e**) Cl 2p of BiOI/BiOCl composite.

To further analyse the surface chemical composition of the BiOI/BiOCl composite, X-ray photoelectron spectroscopy (XPS) were conducted. The XPS survey in Figure 5a shows that BiOI/BiOCl contains major elements of Bi, O, I and Cl as well as a certain amount of C (the adventitious carbon from the XPS instruments [25,31]). In Figure 5b, the two peaks with the binding energies of 158.67 and 163.99 eV are attributed to Bi 4f_7/2_ and Bi 4f_5/2_, respectively, which represent the typical Bi^3+^ in BiOI/BiOCl composite. The high-resolution O 1s scan is presented in Figure 5c. The dominant peak at 529.63 eV can be assigned to the lattice oxygen in the (BiO)_2_^2+^ slabs of the BiOI/BiOCl layered structure, and the other peak at 532.49 eV may be attributed to the surface hydroxyl groups [32]. The peaks of I 3d in Figure 5d can be found at 630.02 and 618.53 eV, which could be attributed to I 3d_3/2_ and I 3d_5/2_, respectively, corresponding to I^−^ in the BiOI/BiOCl composite. The high-resolution scan of Cl 2p is shown in Figure 5e with one peak centered at 197.47 eV, which is ascribed to Cl 2p_3/2_. The overall surface chemical compositions including atomic concentrations of the major elements are listed in Table 1. It is noted that the atomic ratio of I/Cl on the surface of the composite catalyst is about 2.9:1, which is much lower than 9:1 applied during the catalyst preparation, indicating a high concentration of chlorine ions on the surface.

### 3.4. Specific Surface Areas and Pore Structure

**Figure 6:**
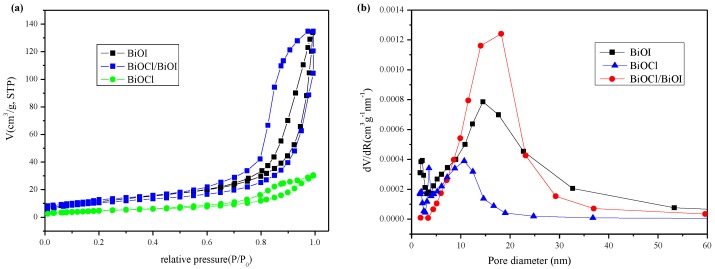
(**a**) Nitrogen sorption isotherms of BiOI, BiOCl, and BiOI/BiOCl; (**b**) Pore size distributions of BiOI, BiOCl, and BiOI/BiOCl.

A large specific surface area of a photocatalyst is beneficial to the enhancement of the photocatalytic performance. Nitrogen adsorption/desorption isotherms and the pore size distribution of the BiOI, BiOCl, and BiOI/BiOCl composites (with a mole ratio of I^−^:Cl^−^ = 9:1) are estimated as shown in Figure 6a,b. The isotherms of all three samples fall into type IV isotherms with a distinct hysteresis loop observed in the range of 0.6–1.0 *P*/*P*_0_, suggesting the formation of capillary condensation related to mesopores between closely-packed spherical particles [33]. The presence of a small amount of Cl^−^ in the BiOI exerted obvious influence on the Brunauer-Emmett-Teller (BET) surface areas and pore structure (Table 2). The BET surface area decreases a little from 42.5 to 37.7 m^2^·g^−1^ with the average pore size increased from 15.7 to 16.8 nm. Both of the isotherms of BiOI and BiOI/BiOCl manifest much higher adsorption capacity than BiOCl at high relative pressure (*P*/*P*_0_ in the range of 0.8–1.0), indicating that larger inter-aggregated pores were generated and became predominant with larger total pore volumes and a higher adsorption capacity, due to the aggregation of sheet-like nanoparticles [24]. This could be confirmed by the SEM and TEM images with the flower-like 3D microstructure interwoven by very thin nanoplates.

Compared with pure BiOI, the BiOI/BiOCl composites also exhibited a larger hysteresis loop with a similar shape for the isotherms. In addition, the pore-size distribution of BiOI/BiOCl composites became more uniform. The pure BiOI sample contains a bimodal mesopore size distribution from ca. 2.1 nm to ca. 14.7 nm. In contrast, the larger mesopores in the BiOI/BiOCl composites occupied the main portion of the total pore volume with a maximum pore diameter of 18.2 nm, which may be produced from the inter-aggregated secondary particles and the stack of nanoplates.

### 3.5. Photocatalytic Degradation of MO

#### 3.5.1. Adsorption of MO

**Figure 7:**
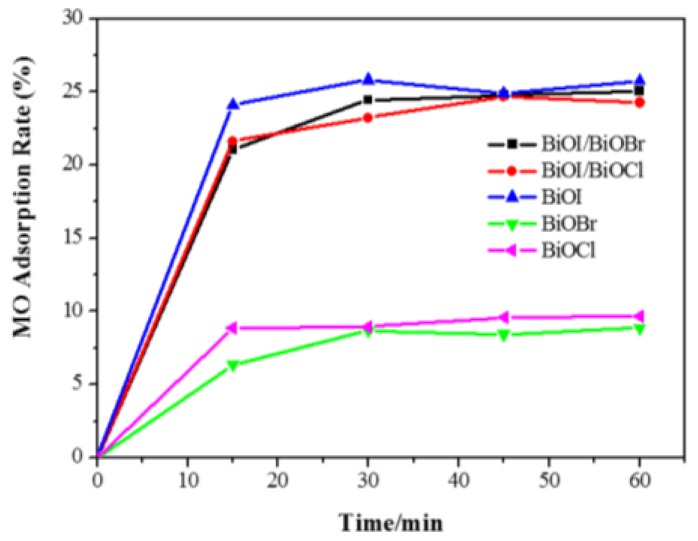
Adsorbed MO in the dark for BiOX (X = Cl, Br, I) and BiOI/BiOX (X = Br, Cl) composites (*C*_0_ = 20 ppm, catalyst dosage = 0.2 g/L).

All the adsorption experiments were conducted in the dark with an initial concentration of 20 ppm MO and a catalyst dosage of 0.2 g/L [34]. As shown in Figure 7, both BiOCl and BiOBr achieved adsorption equilibrium after 30 min with 10% dye adsorption. The pure BiOI and the composites (BiOI/BiOX, X = Br, Cl) exhibited a higher adsorption capacity of about 25% dye removal in 30 min.

Pure BiOI shows the highest adsorption rate of 25.7% MO removal after 60 min, which could be due to the highest BET surface area among the photocatalysts. The greater dye adsorption rate would contribute to faster dye degradation as the larger surface area would be more favourable for dye molecules to adsorb onto the active sites of the photocatalyst, giving rise to an enhanced photocatalytic activity. Overall, when 0.05 g of catalyst was introduced into the 20 ppm methyl orange solution, the adsorption removal efficiencies of all of the catalysts are less than 30% after adsorption equilibrium in 30 min. In this case, adsorption will not exhibit a considerable influence on the investigation of photodegradation efficiency.

#### 3.5.2. Comparison of Photocatalytic Activity

**Figure 8:**
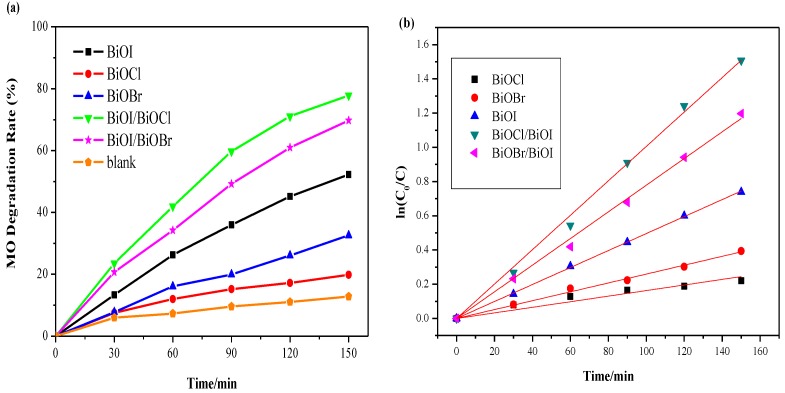
(**a**) Comparison of photodegradation efficiencies of different samples under visible irradiation; (**b**) pseudo-first-order kinetics curves of methyl orange (MO) degradation over different samples under visible irradiation.

**Figure 9:**
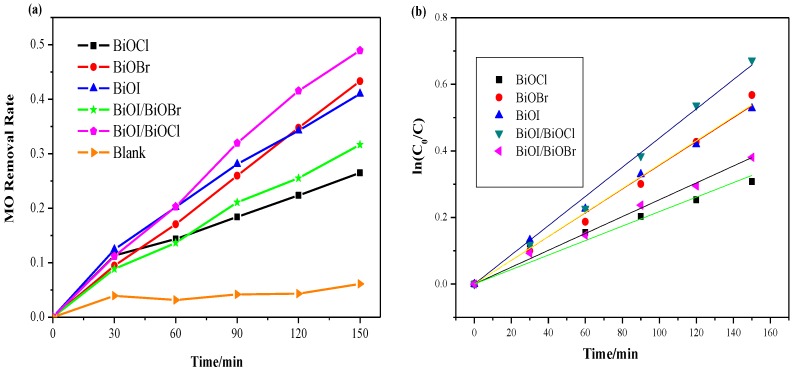
(**a**) Comparison of photodegradation efficiencies of different samples under UV irradiation; (**b**) pseudo-first-order kinetics curves of MO degradation over different samples under UV irradiation.

All the photocatalysts were tested under both visible and UV irradiations as shown in Figure 8 and Figure 9. Figure 8a demonstrates that only 12% of MO was removed after 2.5 h for visible-light irradiation without any catalyst, suggesting that MO is chemically stable and refractory to decomposition by photolysis. Both of the composite semiconductors BiOI/BiOCl and BiOI/BiOBr had higher photocatalytic activity than pure BiOI under visible-light irradiation. Among all the catalysts, the BiOI/BiOCl composite gave 78% MO removal after 150 min, which was 25% higher than the pure BiOI. The lattice of single BiOI was expanded by the coupling of BiOX (X = Cl, Br), leading to the recombination between the lattices to form a three-dimensional spherical structure with heterojunction interfaces, which will contribute to the efficient separation of photogenerated electron-hole pairs. As shown in Figure 4, the band edges of both pure BiOI and BiOI/BiOX (X = Cl, Br) fall into the visible light region, with the composites showing even higher light absorption capacity than the pure BiOI. The photocatalytic activity enhancement of BiOI/BiOX (X = Cl, Br) could be ascribed to the retardation of electron-hole recombination with improved interfacial charge transfer efficiency as well as a stronger light absorption ability.

Relative to BiOX (X = Cl, Br), BiOI has a narrow band gap of around 1.9 eV, which can be excited by visible light with a more efficient utilization of solar energy. Additionally, BiOI and its composite (BiOI/BiOCl) with larger specific surface areas (SSAs) can not only supply more active sites for pollutant degradation but also promote the separation of the electron-hole pairs [35]. Nevertheless, BiOCl is severely limited for visible light adsorption due to its wide bandgap (3.25 eV) which caused a poor catalytic performance in the visible light region. In Figure 8, BiOCl exhibits the lowest photodegradation activity among all the catalysts under visible light irradiation. According to pseudo-first-order kinetics [36], the apparent pseudo-first-order rate constants (*k_app_*) were obtained as a comparative parameter for the photocatalytic activity of different catalysts.

(1) $$\ln(\frac{C_{0}}{C})=kKt=k_{\mathrm{app}}t\;\mathrm{or}\;C_{t}=C_{0}e^{-k_{\mathrm{app}}t}$$

where *C*_0_ is the original concentration of the dyes and *C* is the concentration at the reaction time *t*; *k* is the reaction rate constant; *K* is the adsorption coefficient of the reactant. The linear time dependence of ln(*C*_0_/*C*) is plotted in Figure 8b and Figure 9b correspondingly.

Based on the curves from Figure 8b and Figure 9b, the calculated values of *k_app_* and *R*^2^ are displayed in Table 3. As observed in Figure 9b and Table 3, the BiOI/BiOCl composite demonstrates the highest photodegradation efficiency under UV light irradiation with almost 50% MO removal in 150 min, compared with the pure BiOI catalyst of 40%. Given the performances under visible light irradiation, BiOBr turns out to be an excellent UV responsive photocatalyst, as BiOBr shows good UV light absorption capacity according to Figure 4. The BiOI/BiOBr composites present an outstanding photocatalytic activity under visible light irradiation, yet an inferior activity under UV light irradiation. In summary, BiOI/BiOCl exhibited the highest photocatalytic performance both under UV and visible light irradiation, indicating that the intimate interaction between BiOI and BiOCl is crucial for the formation of a charge-separation heterojunction [37]. However, BiOCl shows the lowest activity under both UV and visible irradiations, due to the poor light-absorption capability and a low specific surface area.

**Figure 10:**
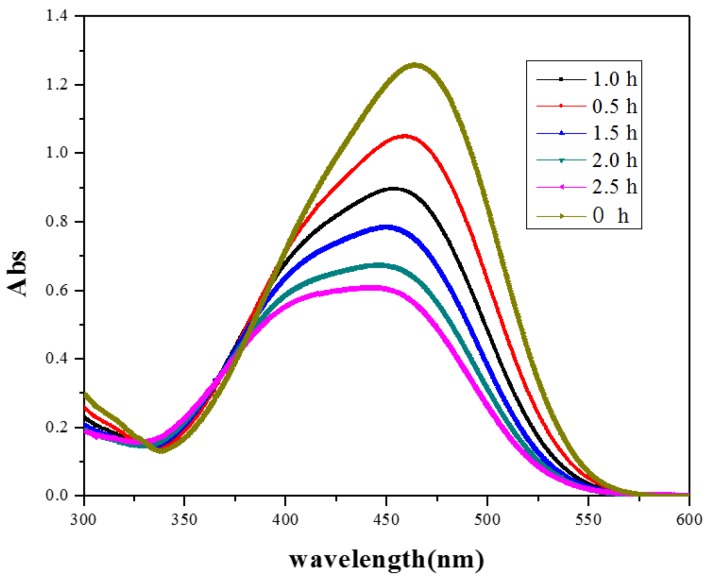
Temporal evolution of the spectra during the photodegradation of MO mediated by BiOI/BiOCl composite.

Figure 10 shows a typical UV-Vis absorption spectrum of the MO solution during degradation by the BiOI/BiOCl composite at different time intervals. As the irradiation proceeded, the absorption peak at 465 nm shows a blue-shift and becomes broadened simultaneously, implying that the decomposition of MO molecules is due to the demethylation reaction in which the methyl group is substituted by a hydrogen atom after the homolytic breaking of the nitrogen–carbon bond [38].

**Figure A1:**
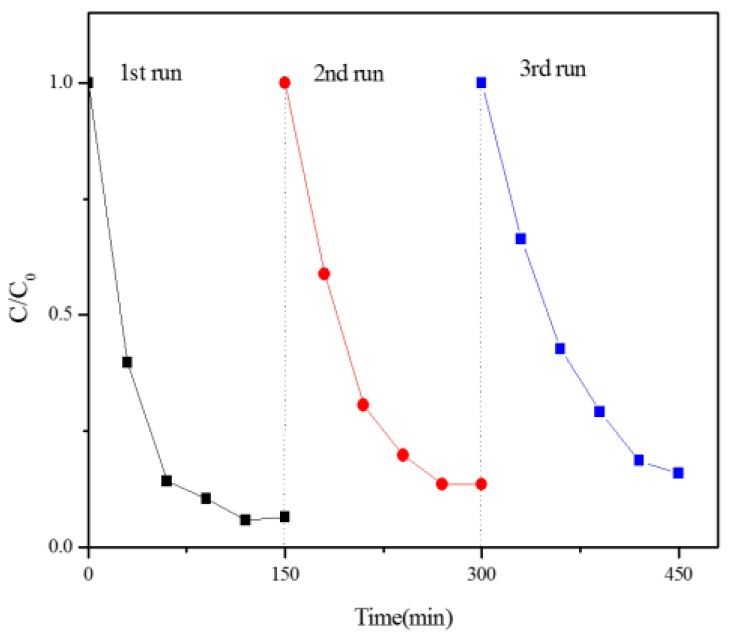
Cycling runs in the photocatalytic degradation of MO with BiOI/BiOCl as the photocatalyst under visible light irradiation.

Except for high photocatalytic activity, the lifetime of the photocatalyst is also a key parameter for the practical application of the catalytic process. To observe sample stabilities, recycling experiments were conducted with the BiOI/BiOCl composite photocatalyst to degrade MO under visible light irradiation using an MSR 575/2 metal halide lamp (575 W, Philips, Somerset, NJ, USA) with the intensity at 250 mW/cm^2^ (400–1050 nm). As shown in Figure A1, the photocatalytic degradation was 93.5%, 86.6%, and 84.2% for the first, second, and third run, respectively. Thus, the sample exhibits good stability without a remarkable decline of photocatalytic activity.

**Figure 11:**
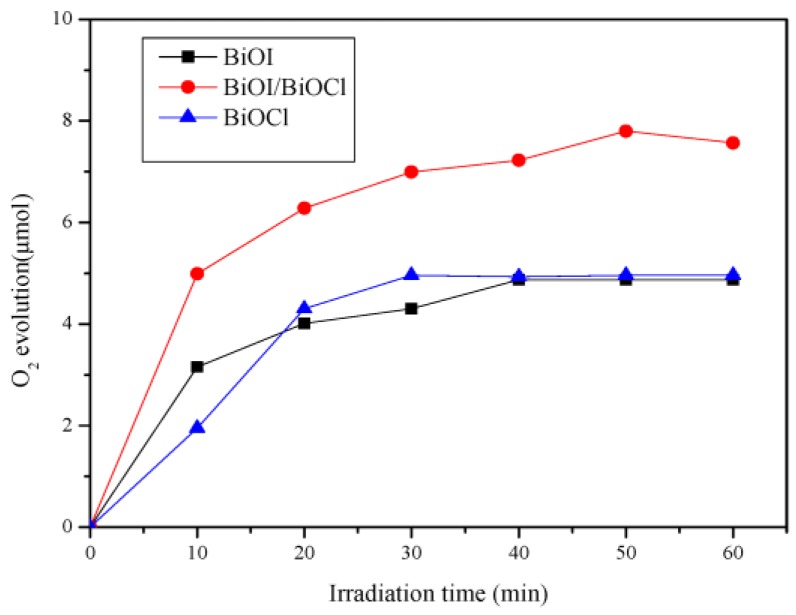
The photocatalytic O_2_ evolution of BiOI, BiOCl, and BiOI/BiOCl composites.

According to the previous studies, the valence band energies of BiOI and BiOCl were calculated to be 2.42 and 3.44 eV, respectively [24,25], both of which are more positive than the standard redox potential of H_2_O/O_2_ (1.23 eV vs. reversible hydrogen electrode (RHE) at pH = 0) [39]. Theoretically, both BiOI and BiOCl including their composites can oxidize H_2_O to produce O_2_. Therefore, BiOI/BiOCl with the highest photocatalytic activity was chosen to explore the potential for photocatalytic water oxidation. Figure 11 describes the time dependence of O_2_ evolution from water over the synthesized catalysts. As can be seen, the O_2_ evolution rate is 7.57 μmol/h for BiOI/BiOCl, which is 1.55 and 1.53 times higher than that of pure BiOI (4.87 μmol/h) and BiOCl (4.96 μmol/h) accordingly after 1 h of simulated solar light irradiation. It is also observed that the water oxidation rates rapidly speeded up in the first 20 min, then went through a plateau afterwards. This result indicates that an effective heterojunction has been constructed between BiOI and BiOCl, which leads to an enhancement of charge transfer and separation efficiency.

#### 3.5.3. Photocatalytic Mechanism of the BiOI/BiOCl Composite

**Figure 12:**
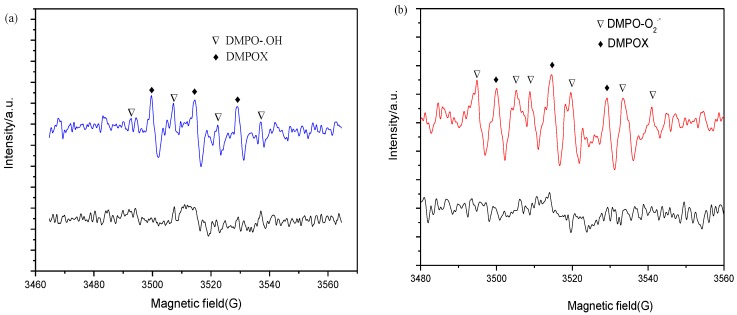
Electron paramagnetic resonance (EPR) spectra of hydroxyl (**a**) and superoxide (**b**) radicals over the BiOI/BiOCl sample under solar light irradiation (DMPOX was denoted as oxidized 5,5-dimethyl-1-pyrroline N-oxide (DMPO)).

**Figure 13:**
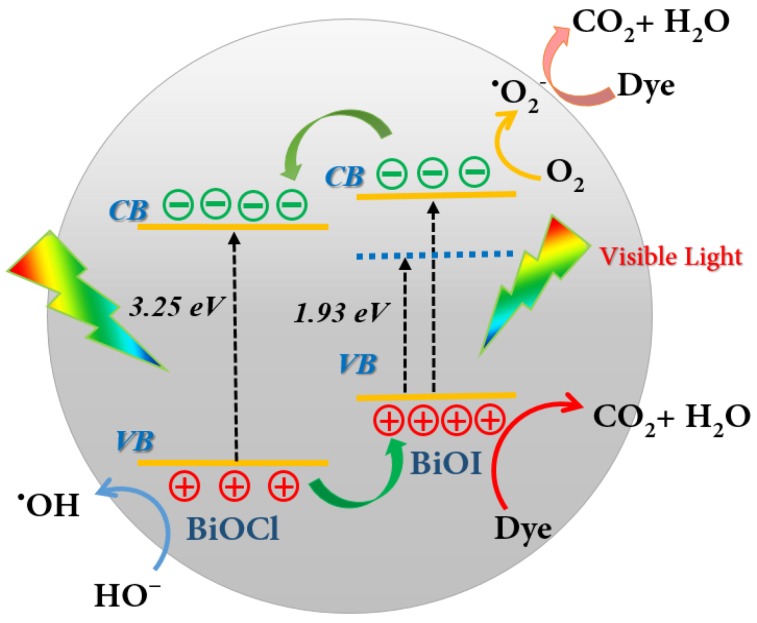
Proposed mechanistic diagram of the photocatalytic process for the BiOI/BiOCl photocatalyst under visible light irradiation.

Given the results above, it could be concluded that the photocatalytic performance of BiOI is highly promoted after coupling with BiOCl to form an effective heterojunction. To further explore the in-depth mechanism, in situ electron paramagnetic resonance (EPR) technology was used to probe the reactive oxygen species in the photodegradation. Both hydroxyl (·OH) and superoxide radicals (·O_2_^−^) were detected with characteristic peaks as shown in Figure 12. The 5,5-dimethyl-pyrrolidone-(2)-oxyl-(1) (DMPOX) represents oxidized DMPO impurities due to a long-time irradiation. In Figure 12a, the weak signals of hydroxyl radicals (·OH) were observed after irradiation for 5 min, indicating that the photo-generated holes might combine with adsorbed H_2_O molecules to produce a small amount of ·OH. Figure 13b revealed that the characteristic peaks of superoxide radicals (·O_2_^−^) with strong intensities were detected, indicating that the superoxide radicals could be the main reactive oxygen species during the photocatalytic reaction process. According to recent studies [40,41,42,43], surface peroxo species could be formed via the disproportionation of the superoxide radicals or coupling of hydroxyl radicals, and become the primary intermediates of photocatalytic reactions, which will be involved in the photocatalytic reaction processes including photodegradation of organic pollutants and oxygen evolution. The detailed mechanism involving peroxo species requires further exploration in future studies.

According to the semi-empirical equation (Equation (2)) based on the Mulliken electronegativity theory [44], both the conduction band potential (CB) and valence band potential (VB) of BiOI and BiOCl were calculated.

(2) $$E_{\mathrm{VB}}=X-E_{e}+0.5\times E_{g}$$

where *E_g_* is the band gap potential, *E_e_* is the energy of free electrons on the hydrogen scale (4.5 eV), and *X* is the absolute electronegativity of the constituent atoms. 

According to the UV-Vis diffuse spectra, the values of *E_g_* of pure BiOCl and BiOI were calculated to be 3.25 and 1.93 eV, respectively. The calculated valence band (VB) positions of BiOCl and BiOI were estimated to be 3.44 and 2.39 eV accordingly. Thus, the conduction band (CB) positions of BiOCl and BiOI were obtained to be 0.19 and 0.46 eV.

Based on analysis and discussion above, a schematic illustration was proposed to unravel the formation of the BiOI/BiOCl heterojunction structure in Figure 13. Under visible light irradiation, only BiOI with a narrow band gap of 1.93 eV could be excited, with photo-generated electrons in the valence band being excited to a higher potential edge than the original one to form a new conduction band [27], which is even higher than the CB of BiOCl. According to energy band structure theory, electrons from the CB of BiOI will be transferred to the lower lying CB of BiOCl to generate an electron center; meanwhile, the holes from the VB of BiOCl will be transferred oppositely to the VB of BiOI to create a hole center. Herein, the photo-excited electrons and holes could be efficiently separated with a lengthened lifetime. Moreover, since the new conduction band of BiOI is more negative than the reduction potential of O_2_/·O_2_^−^ (−0.33 eV) [16], the oxygen molecules could be reduced to ·O_2_^−^ radicals by the electrons, which is further supported by the EPR results. Compared with the standard reduction potential of ·OH/H_2_O (2.27 eV) or ·OH/OH^−^ (2.38 eV) [16], the VB potential of BiOI is 2.39 eV, indicating that oxidative holes (h^+^) on the surface of BiOI experienced difficulty in directly oxidizing H_2_O or OH^−^ into ·OH. Instead, most of the h^+^ would react with dye molecules directly and synergistically promote the dye decomposition. However, the VB potential of BiOCl is 3.44 eV, which is positive enough to oxidize H_2_O or OH^−^ into ·OH radicals. Thus, the small amount of h^+^ left in the VB of BiOCl could produce some hydroxyl radicals, contributing to the dye degradation as well. In summary, the unique heterojunction between BiOI and BiOCl could render the charge carrier separation and transport more efficiently, which are the key factors for improved photocatalytic performance, compared to the pure BiOI. The strong light absorption capacity of the BiOI/BiOCl composite as well as the enlarged specific surface area also account for the excellent photodegradation efficiency.

#### 3.5.4. Photodegradation of Dyes under Natural Solar Light Irradiation

**Figure 14:**
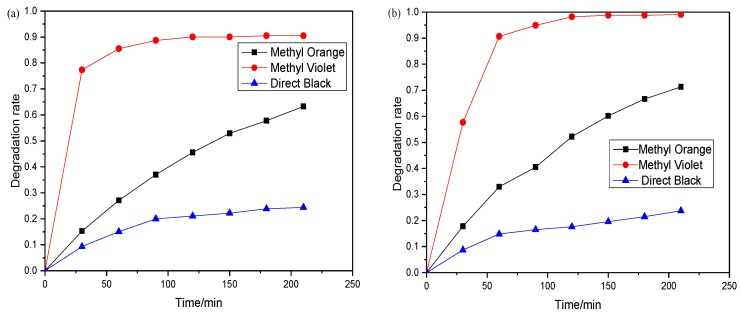
Photocatalytic degradation of typical dyes under natural solar light irradiation with (**a**) BiOI and (**b**) BiOI/BiOCl.

To further investigate the practical application of the optimal photocatalysts, two other typical dyes of methyl violet (MV) and direct black (DB) together with methyl orange were studied for the estimation of the photodegradation effectiveness of the BiOI and BiOI/BiOCl composites under natural solar light irradiation. All of the experiments were performed simultaneously outdoor (Nanjing, China) at noon (11:30 a.m.–3:00 p.m.) in the summer season of June, under the same conditions listed in Section 3.5.2 except for the light source. The degradation efficiency results are depicted in Figure 14. Among the three dye pollutants, methyl violet is most vulnerable to sunlight irradiation on the catalysts. 100% MV degradation could be achieved in 210 min under natural sunlight irradiation on the BiOI/BiOCl composite. Moreover, the degradation of methyl violet attained a superior reaction rate in the first 60 min. Overall, the degradation rate was enhanced by using BiOI/BiOCl as the photocatalyst rather than the pure BiOI for MO and MV. Based on the analysis of the molecular structure of the three dyes, it could be deduced that the degradation efficiency of the dyes was closely related to the azo functional groups in the molecular structure. All the dye molecular structures are listed in Table A1. Zero, one, and three azo groups were functionalized in methyl violet, methyl orange, and direct black, respectively, which might account for the reason that direct black is refractory to be decomposed on both the BiOI and BiOI/BiOCl catalysts. 

## 4. Conclusions

In summary, flower-like 3D BiOI/BiOX (X = Br or Cl) hybrids have been successfully fabricated via a facile one-pot solvothermal approach. The BiOI/BiOCl hybrids present fluffy and porous 3D microspheres with large specific surface areas and high light absorption abilities. Under visible light irradiation, both of the composites exhibited significant enhancement of the photocatalytic oxidation performance compared to pure BiOI. The apparent reaction rate for MO degradation is 2.1 times higher over BiOI/BiOCl, and 1.6 times higher over BiOI/BiOBr than that of pure BiOI. Moreover, BiOI/BiOCl demonstrated a slight promotion under UV light irradiation, which is 1.3 times higher than pure BiOI. Moreover, the BiOI/BiOCl composite also displayed excellent water oxidation ability with enhanced O_2_ evolution from the water. The enhancement of photocatalytic activity could be attributed to the formation of a heterojunction between BiOI and BiOCl, which facilitates the separation and transportation of charge carriers more efficiently with a rationally-engineered energy band structure. In addition, the nanoporous structure, larger specific surface area, and the stronger light absorption capacity both in the visible and UV region also contributed to the excellent photocatalytic activity of the BiOI/BiOCl composites. The photodegradation was evidenced to be ascribed to the superoxide radicals, oxidative holes, and a minor amount of hydroxyl radicals. This study deepens the understanding of BiOI/BiOCl composites for enhanced photodegradation and water oxidation. The rational design of hybrid materials in photocatalysis will provide promising candidates for further applications in photocatalysis and solar energy conversion.

## Figures and Tables

**Table 1:** Surface chemical composition and concentration of the BiOI/BiOCl catalyst.

Element	O 1s	Cl 2p	Bi 4f	I 3d
Atomic %	30.57	2.44	22.03	7.09

**Table 2:** The Brunauer-Emmett-Teller (BET) surface areas and pore structures of the photocatalysts.

Catalyst	Surface Area/m^2^·g^−1^	Pore Volume (cm^3^·g^−1^)	Pore Size (nm)
BiOI	42.4	0.20	15.7
BiOCl	17.0	0.046	8.8
BiOCl/BiOI	37.7	0.20	16.8

**Table 3:** Photodegradation rate constants of various catalysts under UV and visible light.

Light Irradiation	Photocatalysts	*K_app_* (min^−1^)	*R*^2^
UV	BiOCl	0.0022	0.979
	BiOBr	0.0036	0.996
	BiOI	0.0036	0.998
	BiOI/BiOBr	0.0025	0.998
	BiOI/BiOCl	0.0044	0.998
Visible	BiOCl	0.0016	0.976
	BiOBr	0.0026	0.998
	BiOI	0.0050	0.999
	BiOI/BiOBr	0.0078	0.999
	BiOI/BiOCl	0.0100	0.999

## Appendix A

**Table A1:** Molecular structures and characteristics of the dyes.

Dye	Molecular Structure	Application	Safety
Methyl Orange		pH indicator	mutagenicproperties
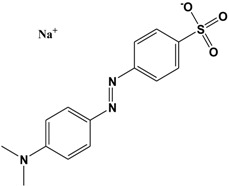
Methyl Violet		purple dye for textiles	mutagen and mitotic poison
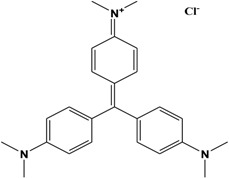
Direct Black		silk dyeing/printing/leather shading	carcinogenicity andreproductive toxicity
